# Supplementary material for: Supportive Care Needs of Patients with Breast Cancer Who Self-Identify as Black: An Integrative Review
Source: Curr Oncol. 2025 Oct 18;32(10):580. doi: 10.3390/curroncol32100580 (PMC12564611; doi:10.3390/curroncol32100580)
Supplement: Supplementary file 1 [file curroncol-32-00580-s001.zip › Supplementary Table S1.pdf]

Supplemental Table S1. Search Strategy

Breast Cancer	Black Identity	Supportive Care/Navigation
• Breast carcinoma* mp.kw<br>• Breast neoplasm* mp.kw<br>• Breast tum* mp.kw<br>• "Breast oncology" mp.kw	• "African American" mp.kw<br>• Black mp.kw<br>• "Black African" mp.kw<br>• Caribbean mp.kw<br>• Afrocentric mp.kw	• Psychosocial mp.kw<br>• "Supportive care" mp.kw<br>• "Health services" mp.kw<br>• Supports mp.kw<br>• "Patient?cent* care" mp.kw<br>• "Patient-cent* care" mp.kw<br>• Psycho-oncology mp.kw<br>• Navigation mp.kw<br>• "Peer support" mp.kw<br>• "Health promotion" mp.kw<br>• Education mp.kw
