# Supplementary material for: Supportive Care Needs of Patients with Breast Cancer Who Self-Identify as Black: An Integrative Review
Source: Curr Oncol. 2025 Oct 18;32(10):580. doi: 10.3390/curroncol32100580 (PMC12564611; doi:10.3390/curroncol32100580)
Supplement: Supplementary file 1 [file curroncol-32-00580-s001.zip › Supplementary Table S2.pdf]

Supplementary Table S2. Details of retained articles

First Author Last Name	Year of Publication	Citation	Spiritual support	Psychosocial	Peer Support	Child/Family Supports	Caregiver Support	Culturally Relevant Care	Quality of Patient-Physician Care	Financial Supports	Transportation	Access to Resources	Racialized Data	Fertility Preservation	Navigation	Fitness & Nutrition	Body Image	Sexual Health	Career and employment	Survivorship Transition
Adam	2017	Health Commun. 2021;36(11):1331-1342.	1	1	1	1	0	0	0	0	0	1	0	0	1	0	0	0	0	0
Anderson	2021	Health Commun. 2021;36(11):1331-1342.	0	0	0	0	0	1	1	0	0	1	1	0	1	0	0	0	0	0
Ashing-Giwa	1997	J Psychosocial Oncology. 1997;15(2): 19-35.	1	1	1	1	0	1	1	1	1	1	0	0	0	0	0	0	1	0
Ashing-Giwa	2013	J Cancer Surviv. 2013;7(3):283-91.	1	1	0	0	0	1	0	1	0	1	0	0	0	0	0	0	0	0
Barsevick	2016	Support Care Cancer. 2016;24(12):4979-4986.	1	1	0	0	0	0	0	0	0	1	0	1	0	0	1	1	0	0
Black	2020	Qual Health Res. 2020;30(13):2019-2032.	0	0	1	0	0	0	0	0	0	0	0	1	0	0	1	1	0	0
Black	2018	Support Care Cancer. 2018;26(1):231-240.	1	1	0	0	0	0	0	1	0	1	0	0	0	1	1	0	0	0
Ceballos	2021	J Psychosoc Oncol. 2021;39(1):35-53.	0	0	1	0	0	1	1	0	0	1	0	0	0	0	0	0	0	1
Christian	2017	Patient Educ Couns. 2017;100(7):1360-1366.	0	1	0	0	0	0	0	0	0	1	0	0	1	1	1	0	0	1
Coggin	2006	J Psychosoc Oncol. 2006;24(4):107-22.	1	0	0	1	0	0	0	0	0	1	0	0	0	0	0	0	0	0
Davey	2010	J Psychosoc Oncol. 2010;28(6):683-98.	0	0	1	1	0	0	1	1	0	0	0	0	1	0	0	0	0	0
Davis	2009	Soc Work Health Care. 2009;48(6):561-78.	0	0	1	1	0	0	0	0	0	1	0	0	1	0	0	0	0	1
Davis	2013	Soc Work Health Care.	0	0	0	0	0	0	0	0	0	1	0	0	1	0	0	0	0	0

		2013;52(5):434-48.																		
Felder	2019	Ethn Health. 2019;24(7):737-753.	1	1	1	0	0	0	0	0	1	1	0	0	0	0	0	0	0	1
Ford	2017	J Best Practices in Health Professions Diversity. 2017; 10(1): 28-40.	1	0	1	1	0	0	1	0	0	0	0	0	0	0	0	0	0	0
Fouad	2010	Ethn Dis. 2010;20(2):155-61.	1	1	1	0	0	0	0	1	0	0	0	0	1	0	0	0	0	0
Haynes-Maslow	2916	J Cancer Educ. 2016;31(1):166-71.	0	0	1	0	1	0	0	0	0	1	0	0	1	0	0	0	0	0
Haynes-Maslow	2017	Support Care Cancer. 2017;25(5):1511-1517.	0	1	1	1	0	0	1	0	0	1	0	0	0	0	0	0	0	0
Khubchandani	2025	Ann Surg Oncol. 2025;32(1):104-114.	0	0	1	1	0	0	0	0	0	0	0	0	1	0	0	0	0	0
Knobf	2018	J Psychosoc Oncol. 2018;36(4):406-417.	1	0	1	0	0	0	0	0	0	0	0	0	1	0	0	0	0	0
Ko	2023	Cancer. 2023;129(S19):3087-3101.	1	0	0	0	0	0	1	1	0	1	1	0	0	1	1	0	0	1
Lackey	2001	Oncol Nurs Forum. 2001;28(3):519-27.	1	0	1	0	0	0	0	0	0	0	0	0	0	0	1	1	0	0
Lee	2024	PLoS One. 2024;19(10):e0312547.	0	1	1	0	0	0	1	1	0	0	0	0	1	0	0	0	0	0
Lewis	2012	J Psychosoc Oncol. 2012;30(2):168-84.	1	1	1	1	0	0	0	0	0	0	0	1	1	0	0	1	0	0
Lopez	2005	Qual Health Res. 2005;15(1):99-115.	1	0	1	0	0	0	1	0	0	1	1	0	0	0	0	0	0	0
Mollica	2015	Cancer Nurs. 2015;38(1):16-22.	1	1	0	0	0	0	0	0	0	1	0	0	0	0	0	0	0	1
Moore	2001	Cancer Nurs. 2001;24(1):35-42.	0	0	1	0	0	1	0	0	0	1	0	0	0	0	0	0	0	0
Paxton	2014	J Cancer Surviv. 2014;8(1):31-8.	0	0	0	0	0	0	0	0	0	0	1	0	0	1	0	0	0	0

Placentine	2018	West J Nurs Res. 2018;40(12):1885-1902.	0	0	0	0	0	0	0	0	1	0	0	0	0	1	1	0	0	0
Ragas	2014	Womens Health Issues. 2014;24(5):511-8.	0	0	0	0	0	0	1	1	0	1	0	0	1	1	0	0	0	0
Royak-Schaler	2008	Oncol Nurs Forum. 2008;35(5):836-43.	0	0	0	0	0	0	1	0	0	1	0	0	0	1	0	0	0	1
St. George	2020	Psychooncology. 2020;29(1):182-194.	0	0	1	1	0	0	0	0	0	1	0	0	0	0	0	1	0	0
Thompson	2006	Cancer Nurs. 2006;29(6):478-87.	1	0	1	1	0	0	1	1	0	1	0	0	0	1	0	0	0	1
Wilmoth	2001	Oncol Nurs Forum. 2001;28(5):875-9.	0	1	1	1	0	0	0	0	0	1	0	0	0	1	0	1	0	0
